# Evidence Supporting the Management of Medical Conditions During Long-Duration Spaceflight: Protocol for a Scoping Review

**DOI:** 10.2196/24323

**Published:** 2021-03-29

**Authors:** Kim-Anh Tran, Neal William Pollock, Caroline Rhéaume, Payal Sonya Razdan, Félix-Antoine Fortier, Lara Dutil-Fafard, Camille Morin, David Pierre-Marie Monnot, Maxime Huot-Lavoie, Philippe Simard-Sauriol, Sam Chandavong, Geneviève Le Pabic, Jean-Philippe LeBlanc, Daniel Lafond, Andréanne Marion, Patrick Michel Archambault

**Affiliations:** 1 Faculty of Medicine Université Laval Quebec, QC Canada; 2 Centre de recherche intégrée pour un système apprenant en santé et services sociaux Centres intégrés de santé et de services sociaux de Chaudière-Appalaches Lévis, QC Canada; 3 VITAM - Centre de recherche en santé durable Université Laval Centre intégré universitaire de santé et de services sociaux de la Capitale-Nationale Quebec, QC Canada; 4 Faculty of Science and Engineering Université Laval Quebec, QC Canada; 5 Research and Technology Thales Canada Quebec, QC Canada; 6 Faculty of Medicine Université de Sherbrooke Sherbrooke, QC Canada

**Keywords:** spaceflight, astronauts, microgravity, weightlessness, acute coronary syndrome, arrhythmia, atrial fibrillation, eye penetration, intraocular foreign body, herniated disk, nephrolithiasis, pulmonary embolism, retinal detachment, sepsis, stroke, spaceflight associated neuro-ocular syndrome

## Abstract

**Background:**

Future long-duration space exploration missions, such as traveling to Mars, will create an increase in communication time delays and disruptions and remove the viability of emergency returns to Earth for timely medical treatment. Thus, higher levels of medical autonomy are necessary. Crew selection is proposed as the first line of defense to minimize medical risk for future missions; however, the second proposed line of defense is medical preparedness and crew member autonomy. In an effort to develop a decision support system, the Canadian Space Agency mandated a team of scientists from Thales Research and Technology Canada (Québec, QC) and Université Laval (Québec, QC) to create an evidence-based medical condition database linking mission-critical human conditions with key causal factors, diagnostic and treatment information, and probable outcomes.

**Objective:**

To complement this database, we are currently conducting a scoping review to better understand the depth and breadth of evidence about managing medical conditions in space.

**Methods:**

This scoping review will adhere to quality standards for scoping reviews, employing Levac, Colquhoun, and O’Brien's 6-stage methodology; the reported results will follow the PRISMA (Preferred Reporting Items for Systematic Reviews and Meta-Analyses) extension for scoping reviews. In stage 1, we identified the research question in collaboration with the Canadian Space Agency (CSA), the main knowledge user. We prioritized 10 medical conditions: (1) acute coronary syndrome, (2) atrial fibrillation, (3) eye penetration, (4) herniated disk, (5) nephrolithiasis, (6) pulmonary embolism, (7) retinal detachment, (8) sepsis, (9) stroke, and (10) spaceflight associated neuro-ocular syndrome. In stage 2, with the help of an information specialist from Cochrane Canada Francophone, papers were identified through searches of the following databases: ARC, Embase, IeeeXplore, Medline Ovid, PsychINFO, and Web of Science. In stage 3, studies will be selected and assessed using a 3-step process and emerging, refined exclusion criteria. In stage 4, the data will be charted in a table based on parameters required by the CSA and developed using Google spreadsheets for shared access. In stage 5, evidence-based descriptive summaries will be produced for each condition, as well as descriptive analyses of collected data. Finally, in stage 6, the findings will be shared with the CSA to guide the completion of this project.

**Results:**

This study was planned in December 2018. Stage 1 has been completed. The initial database search strategy with all target conditions combined identified a total of 10,403 citations to review through title and abstract screening and after duplicate removal. We plan to complete stages 2-6 by the beginning of 2021.

**Conclusions:**

This scoping review will map the literature on the management of 10 priority medical conditions in space. It will also enable us to identify knowledge gaps that must be addressed in future research, ensuring successful and medically safe future missions as humankind embarks upon new frontiers of space exploration.

**International Registered Report Identifier (IRRID):**

DERR1-10.2196/24323

## Introduction

Future long-duration space missions lead to increases in communication time delays and disruption, and render emergency returns to Earth for timely medical treatment an impossibility. This is why future long-duration space travel, such as travel to Mars (whose orbital radius and period is very different from Earth’s), will require higher levels of medical autonomy. To enhance space crew members’ autonomy in the management of acute mission-critical events, and to minimize health issues and degradation in space, a decision-aid system must be developed to support astronauts’ medical autonomy. This system would help support the management of crewmembers’ health during a mission, and the planning and development of high-priority medical technologies and capabilities for extended space exploration.

Crew selection is proposed as the first line of defense to minimize medical risk for future missions; however, the second line of defense is medical preparedness and autonomy. Consequently, exploration-class missions should benefit from intelligent medical systems to support the crew in medical diagnosis, monitoring, treatment, and maintenance of clinical skills [1]. Some recent efforts along these lines include methods and algorithms for generating clinical decision rules [2,3], trend analysis for prognosis and health management [4], and work on developing data architecture for a clinical decision-support system for future exploration [5]. In an effort to develop this decision-support system, the Canadian Space Agency mandated a team of scientists from Thales Research and Technology Canada (Québec, QC) and Université Laval (Québec, QC) to create an evidence-based medical condition database linking mission-critical human conditions with key causal factors, diagnostic and treatment information (including knowledge, skills, equipment, and material needed), and probable outcomes [6]. In order to complement this database, we will conduct a scoping review to better understand the depth and breadth of evidence about managing medical conditions in space.

Our specific objectives are to (1) synthesize the current knowledge about managing medical conditions in space and (2) identify knowledge gaps to be addressed with future research and technology development to ensure better planning and support for long-duration space exploration-class missions requiring medical autonomy.

## Methods

### Overview

A scoping review will be conducted to map the management of medical conditions in space, as this research area consists mostly of emerging evidence. This review will be planned and conducted in adherence to standards of quality for scoping reviews, employing the Levac 6-stage methodology [7]; the results will be reported following the PRISMA (Preferred Reporting Items for Systematic Reviews and Meta-Analysis) extension for scoping reviews [8].

### Stage 1: Identifying the Research Question

This stage was completed during the planning of this study with the Canadian Space Agency (CSA), our main knowledge user. Results of this effort are published elsewhere [6]. In summary, our research team prioritized 10 medical conditions among the list of 100 conditions from the Integrated Medical Model Medical Conditions list [9]. This list was initially produced by National Aeronautics and Space Administration (NASA) researchers to establish which medical conditions were most relevant (eg, high likelihood of occurrence) to prepare risk mitigation for future long-duration spaceflight. Using this list, our team applied a set of criteria to help us prioritize the top 10 conditions for which the crew would need enhanced medical autonomy; these criteria were a high risk to the mission, high level of contagion, high likelihood of occurring, a critical treatment time window, different treatment in space, communication frequency, and communication bandwidth. After validating this set of criteria with the CSA, our team produced the final list of 10 target medical conditions, which will be the focus of this scoping review: (1) acute coronary syndrome, (2) atrial fibrillation, (3) eye penetration, (4) herniated disk, (4) nephrolithiasis, (5) pulmonary embolism, (6) retinal detachment, (7) sepsis, (8) stroke, and (9) spaceflight-associated neuro-ocular syndrome (SANS; formerly called visual impairment and intracranial pressure syndrome) [10].

### Stage 2: Identifying Studies and Grey Literature

In collaboration with an information specialist working at Cochrane Canada Francophone, we identified the following scientific databases to be searched: Aerospace Research Central (ARC), Embase, IeeeXplore, Medline Ovid, PsychINFO, and Web of Science. Our search strategy for each database used the following keywords: “astronaut,” “cosmonaut,” “weightlessness,” “space flight,” “spacecraft,” “long-duration space exploration missions,” “space simulation,” “aerospace,” “analog environment,” “deep space,” “ecological system,” “extraplanetary,” “extraterrestrial,” “planets,” “countermeasure,” “United States National Aeronautics and Space Administration,” “aerospace medicine,” “environmental medicine,” “space medicine,” and a series of keywords targeting our 10 selected conditions. These strategies were reviewed and accepted by experts at the CSA and can be found in Multimedia Appendix 1.

Study identification will be supplemented by a grey literature search using the Google search engine and by reviewing reports available through CSA and NASA websites and personal libraries. We will also review documents from included articles’ reference lists to ensure the inclusion of all relevant studies.

### Stage 3: Selecting Literature

#### A 3-Step Evaluation Process

A 3-step process will be used to evaluate publications identified during the previous step and on emerging, refined exclusion criteria. After identifying references from our initial search strategy, we will compile references in a Google spreadsheet, with a unique identification number assigned to each article.

##### Step 1

First, 3 reviewer pairs will independently screen titles and abstracts based on a set of inclusion and exclusion criteria defined through team discussion. The following papers meet the selection criteria and will be retained for the second step of screening: studies about disease events in space, studies about disease events in analog environments (eg, bed rest, head-down tilt, research stations in isolated environments), studies about the incidence or prevalence of disease occurring during space travel or in astronauts (for space or the astronaut cohort from 1990 onward), studies about equipment or protocols developed for space application (from 1985 onward), and studies about diagnostic tests or devices evaluated in space. Papers will be excluded if (1) they were not written in English or French; (2) their content or topic is not relevant to space travel (eg, false keyword identification, technology or protocol only); (3) they did not address the 10 target conditions; (4) they were an editorial, letter to the editor, or abstract only, or they were strictly conceptual, a clinical image piece, or a nonscientific publication; (5) they represented a study about incidence or prevalence of diseases occurring in space or an astronaut cohort published before 1990; (6) the cohort was ill-fit (eg, young children, sick population); (7) the methods were inadequately described (validity unclear); (8) the results were invalid (fatal flaws to the methodology), or (9) the publication was a duplicate. Studies on disease incidence or prevalence occurring in space or astronaut cohorts published before 1990 will not be considered because they are most likely not representative of current epidemiologic factors and crewmembers’ health.

To ensure a consistent application of criteria and to obtain a high level of agreement, screening training sessions between at least one member of each team will be conducted for a first set of approximately 500 citations. Once inclusion and exclusion criteria for the first round are fully understood and applied uniformly between members, the remaining articles will be divided and coded by the team pairs. Each publication will be reviewed by at least 2 members of each team. All disagreements will be resolved through discussions.

##### Step 2

A second round of title and abstract screening will be performed by pairs of reviewers with a new set of refined selection criteria to better define the scope of manuscripts to include in stage 4, when data charting will be performed.

##### Step 3

After narrowing down the article list and better defining this review's final scope for stage 4, pairs of reviewers will proceed to a third and final round of screening, using full-text when available. New, refined exclusion criteria will be determined and then applied after reading included papers from the second round. These new exclusion criteria will be used to decide if data from papers should be charted in stage 4 and included in the final results of the scoping review (stage 5).

### Stage 4: Charting the Data

A data-charting table will be developed using Google spreadsheets for shared access. The table will be based on parameters required by the CSA for its medical conditions parameter database: medical condition name, medical condition category, systematized nomenclature of medicine clinical terms (SNOMED CT) identifier, definition/description of medical condition, incidence/prevalence, risk factors, level of medical knowledge, medical skills, differential diagnosis, history/symptoms, physical findings/signs, imaging, laboratory tests, physiological measurements, psychometric test, pharmacotherapy, nutritional therapy, surgical treatment, physical therapy, medical management/outcomes, support machines, instruments, and disposables. These parameters were selected and refined by reviewers.

The charting table will be trialed for the first 20 studies for refinement as part of an iterative process in which members will update the form until consensus is reached on the final version. The table contained the following parameters to describe each selected paper: a description of the study’s research question (population, intervention, comparison, outcomes, study type), the study subject (human, animal, virtual/theoretical, cadaver, cell culture, infectious pathogen), the study context (space, analog environment, Earth), incidence of disease, the prevalence of disease, the proposed physiopathology in space, risk factors, the odds ratio associated with the risk factor, medical skills for diagnosis, symptoms, physical signs/findings on physical exam, imaging, laboratory tests, physiologic measurements, clinical prediction rules, psychometric tests, pharmaceutical drug treatment, surgical treatment, other treatments (eg, physical therapy, nutritional therapy), medical skills for treatment, medical instruments and equipment, the medical management and outcome, clinical relevance (if it’s a basic science paper), and the study limitations. Definitions for each parameter will be refined by the group to ensure a common understanding and a consistent application during coding and data extraction. Individual reviewers will then extract data for all retained studies included in the third phase. A third reviewer will review discrepancies or disagreements in extraction to resolve them in a group consultation.

### Stage 5: Collating, Summarizing, and Reporting Results

#### Collating and Summarizing

A team of reviewers will summarize the data corresponding to the parameters previously mentioned in the charting stage. This description will map out the literature on selected medical conditions in space. Our analysis will remain descriptive, as publication characteristics and, subsequently, extracted information will be heterogeneous between studies. The publication frequency for each of the 10 target conditions, the type of subjects studied (human vs animal vs theoretical model), and the study context will be reported for each study. The other parameters mentioned in stage 4 will also be presented, and evidence-based descriptive summaries for each target condition will be created. These summaries will contain qualitative and quantitative evidence extracted from selected papers in previous stages.

#### Reporting Results

To present our study results, we will compose a narrative description of the search decision process and a search decision flowchart. Our flowchart will detail results from the search strategy, removal of duplicate references, additions from grey literature and reference checking, and the final number of included publications per medical condition.

To present the results of our data extraction, we will employ descriptive tables and charts. Distribution of publications by medical condition in chart form will help illustrate which medical conditions lacked evidence and should be further investigated. A first table will include the list of retained publications, accompanied by a summary of relevant extracted data on parameters previously mentioned for long-duration deep space exploration. A detailed version of extracted data will be available in appendices. A second table will focus on limitations identified in included studies, either stemming from the methodology or the results. These tables will help knowledge users quickly grasp what is known about the 10 priority medical conditions in space and the current knowledge gaps that need to be addressed in future research directions.

### Stage 6: Consulting Knowledge Users

Prior to conducting this study, the CSA had defined a predetermined set of important parameters for data extraction. During this last stage, we will continue sharing our findings with the CSA to guide the completion of the scoping review. Their feedback will serve as a foundation for future research directions. This stage will enable the CSA to build on the presented results and offer content expertise and perspective to our findings.

## Results

This study was planned in December 2018. Stage 1 has been completed. The initial database search strategy with all target conditions combined identified a total of 10,403 citations to review through title and abstract screening and after duplicate removal. We plan to complete stages 2-6 by the beginning of 2021.

## Discussion

The proposed scoping review will provide an overview of the existing literature on the management of 10 priority medical conditions in space. It will also highlight the knowledge gaps to be filled before international space agencies conduct astronautical missions to Mars. Knowledge gaps may be due to methodological flaws or limitations or simply a lack of primary studies. By identifying the gaps, we believe that it will help direct future high-quality and relevant research addressing areas in need of more primary research. Our work also seeks to synthesize the emerging evidence on 10 target medical conditions in space. We expect our scoping review to synthesize evidence about the diagnostic and therapeutic challenges facing medical crews supporting long-term space missions.

Scientific and technological progress continue to advance, making long-duration spaceflight, such as missions to and from Mars, a possibility in the foreseeable future. Developing a robust, well-equipped spacecraft is not the only requirement necessary for a successful space mission. As highlighted in a review [11] of NASA’s Human Research Program’s priority risks for crew health, spaceflight poses unique health and performance risks, including space radiation, SANS, and nutritional concerns that must be controlled to ensure mission success. Therefore, medical support and autonomy are crucial, as the ability to rapidly evacuate a crewmember to Earth will be impossible. However, administering medical care in space presents multiple challenges that have yet to be resolved.

Space technologies such as satellite and geographic information systems have been applied to global health on Earth [12]; however, the application of Earth-based medical technologies during long-duration space missions has not been widely documented. Extended range missions, such as a trip to Mars, have remained theoretical up to this point. Therefore, we can only hypothesize about medical needs and onboard medical and technological capabilities. This partially explains why space medicine research seems to focus more on predicting and preventing medical events [13] than treating diseases that could occur [14]. Monitoring vital signs with sensors, dosing relevant biomarkers, developing crewmembers’ medical skills, and utilizing artificial intelligence and diagnostic algorithms are all strategies currently being explored by space scientists to help astronauts achieve medical autonomy during deep space missions [14].

Ultimately, we hope our work will help space agencies understand the current possibilities and limits of medical care and management of urgent medical conditions during long-duration spaceflight. This will contribute to informed decisions about the appropriate level of medical training for crew members and the medical equipment and devices needed to ensure diagnosis and treatment in space.

This scoping review will map the literature on the management of 10 priority medical conditions in space. It will also enable us to identify knowledge gaps that must be addressed in future research, ensuring successful and medically safe future missions in humankind’s pursuit of new frontiers of space exploration.

## Appendix

Multimedia Appendix 1 Database search strategies.

## Abbreviations

CSA: Canadian Space Agency
NASA: National Aeronautics and Space Administration
SANS: spaceflight-associated neuro-ocular syndrome
