# Supplementary material for: Evidence Supporting the Management of Medical Conditions During Long-Duration Spaceflight: Protocol for a Scoping Review
Source: JMIR Res Protoc. 2021 Mar 29;10(3):e24323. doi: 10.2196/24323 (PMC8088865; doi:10.2196/24323)
Supplement: Multimedia Appendix 1 [file resprot_v10i3e24323_app1.pdf]

Multimedia Appendix 1: Database Search Strategies

Aerospace Research Central (ARC)

Search strategy ARC 20180731	Hits
“astronaut*” OR "Cosmonaut*"OR "Weightlessness" OR "spacecraft" OR “Space Flight” OR “Long-duration space exploration missions” OR “aerospace” OR “extraterrestrial” OR "United States National Aeronautics and Space Administration" OR “NASA” OR "Space Simulation" OR "aerospace" OR "analog environment*" OR "deep space" OR "extraplanetary" OR "planet*" OR "countermeasure" OR "Weightlessness Countermeasures" OR "ecological system*" OR "Space medicine" (30582 Hits)<br>[Astronaut and space]	
(“astronaut*” OR "Cosmonaut*"OR "Weightlessness" OR "spacecraft" OR “Space Flight” OR “Long-duration space exploration missions” OR “aerospace” OR “extraterrestrial” OR "United States National Aeronautics and Space Administration" OR “NASA” OR "Space Simulation" OR "aerospace" OR "analog environment*" OR "deep space" OR "extraplanetary" OR "planet*" OR "countermeasure" OR "Weightlessness Countermeasures" OR "ecological system*" OR "Space medicine") **AND** ("Atrial fibrillation" OR "atrial flutter")<br>[Atrial fibrillation]	1
(“astronaut*” OR "Cosmonaut*"OR "Weightlessness" OR "spacecraft" OR “Space Flight” OR “Long-duration space exploration missions” OR “aerospace” OR “extraterrestrial” OR "United States National Aeronautics and Space Administration" OR “NASA” OR "Space Simulation" OR "aerospace" OR "analog environment*" OR "deep space" OR "extraplanetary" OR "planet*" OR "countermeasure" OR "Weightlessness Countermeasures" OR "ecological system*" OR "Space medicine") **AND** ("myocardial infarction" OR "heart infarction")<br>[myocardial infarction]	4
(“astronaut*” OR "Cosmonaut*"OR "Weightlessness" OR "spacecraft" OR “Space Flight” OR “Long-duration space exploration missions” OR “aerospace” OR “extraterrestrial” OR "United States National Aeronautics and Space Administration" OR “NASA” OR "Space Simulation" OR "aerospace" OR "analog environment*" OR "deep space" OR "extraplanetary" OR "planet*" OR "countermeasure" OR "Weightlessness Countermeasures" OR "ecological system*" OR "Space medicine") **AND** ("Pulmonary embolism" OR "Pulmonary Thromboembolism")<br>[Pulmonary embolism]	1

(“astronaut*” OR "Cosmonaut*"OR "Weightlessness" OR "spacecraft" OR “Space Flight” OR “Long-duration space exploration missions” OR “aerospace” OR “extraterrestrial” OR "United States National Aeronautics and Space Administration" OR “NASA” OR "Space Simulation" OR "aerospace" OR "analog environment*" OR "deep space" OR "extraplanetary" OR "planet*" OR "countermeasure" OR "Weightlessness Countermeasures" OR "ecological system*" OR "Space medicine") **AND** ("Nephrolithiasis" OR "renal colic" OR "Urolithiasis")<br>[Nephrolithiasis]	8
(“astronaut*” OR "Cosmonaut*"OR "Weightlessness" OR "spacecraft" OR “Space Flight” OR “Long-duration space exploration missions” OR “aerospace” OR “extraterrestrial” OR "United States National Aeronautics and Space Administration" OR “NASA” OR "Space Simulation" OR "aerospace" OR "analog environment*" OR "deep space" OR "extraplanetary" OR "planet*" OR "countermeasure" OR "Weightlessness Countermeasures" OR "ecological system*" OR "Space medicine") **AND** ("retinal detachment")<br>[retinal detachment]	1
(“astronaut*” OR "Cosmonaut*"OR "Weightlessness" OR "spacecraft" OR “Space Flight” OR “Long-duration space exploration missions” OR “aerospace” OR “extraterrestrial” OR "United States National Aeronautics and Space Administration" OR “NASA” OR "Space Simulation" OR "aerospace" OR "analog environment*" OR "deep space" OR "extraplanetary" OR "planet*" OR "countermeasure" OR "Weightlessness Countermeasures" OR "ecological system*" OR "Space medicine") **AND** ("sepsis" OR "septicemia")<br>[sepsis]	5
(“astronaut*” OR "Cosmonaut*"OR "Weightlessness" OR "spacecraft" OR “Space Flight” OR “Long-duration space exploration missions” OR “aerospace” OR “extraterrestrial” OR "United States National Aeronautics and Space Administration" OR “NASA” OR "Space Simulation" OR "aerospace" OR "analog environment*" OR "deep space" OR "extraplanetary" OR "planet*" OR "countermeasure" OR "Weightlessness Countermeasures" OR "ecological system*" OR "Space medicine") **AND** ("stroke" OR "cerebrovascular disorders")<br>[Stroke]	859
(“astronaut*” OR "Cosmonaut*"OR "Weightlessness" OR "spacecraft" OR “Space Flight” OR “Long-duration space exploration missions” OR “aerospace” OR “extraterrestrial” OR "United States National Aeronautics and Space Administration" OR “NASA” OR "Space Simulation" OR "aerospace" OR "analog environment*" OR "deep space" OR "extraplanetary" OR "planet*" OR "countermeasure" OR "Weightlessness Countermeasures" OR "ecological system*" OR "Space medicine") **AND** ("Intervertebral Disc" OR "herniated disk")<br>[Intervertebral Disc]	6

(“astronaut*” OR "Cosmonaut*"OR "Weightlessness" OR "spacecraft" OR “Space Flight” OR “Long-duration space exploration missions” OR “aerospace” OR “extraterrestrial” OR "United States National Aeronautics and Space Administration" OR “NASA” OR "Space Simulation" OR "aerospace" OR "analog environment*" OR "deep space" OR "extraplanetary" OR "planet*" OR "countermeasure" OR "Weightlessness Countermeasures" OR "ecological system*" OR "Space medicine") **AND** ("Cardiogenic shock")<br>[Cardiogenic shock]	0
(“astronaut*” OR "Cosmonaut*"OR "Weightlessness" OR "spacecraft" OR “Space Flight” OR “Long-duration space exploration missions” OR “aerospace” OR “extraterrestrial” OR "United States National Aeronautics and Space Administration" OR “NASA” OR "Space Simulation" OR "aerospace" OR "analog environment*" OR "deep space" OR "extraplanetary" OR "planet*" OR "countermeasure" OR "Weightlessness Countermeasures" OR "ecological system*" OR "Space medicine") **AND** (("Vision Disorders" OR "VIIP") AND ("intracranial pressure"))<br>[Vision Disorders]	2
(“astronaut*” OR "Cosmonaut*"OR "Weightlessness" OR "spacecraft" OR “Space Flight” OR “Long-duration space exploration missions” OR “aerospace” OR “extraterrestrial” OR "United States National Aeronautics and Space Administration" OR “NASA” OR "Space Simulation" OR "aerospace" OR "analog environment*" OR "deep space" OR "extraplanetary" OR "planet*" OR "countermeasure" OR "Weightlessness Countermeasures" OR "ecological system*" OR "Space medicine") **AND** ("Penetrating Eye Injuries" OR "Eye Foreign Bodies")<br>[Penetrating Eye Injuries]	0
(“astronaut*” OR "Cosmonaut*"OR "Weightlessness" OR "spacecraft" OR “Space Flight” OR “Long-duration space exploration missions” OR “aerospace” OR “extraterrestrial” OR "United States National Aeronautics and Space Administration" OR “NASA” OR "Space Simulation" OR "aerospace" OR "analog environment*" OR "deep space" OR "extraplanetary" OR "planet*" OR "countermeasure" OR "Weightlessness Countermeasures" OR "ecological system*" OR "Space medicine") **AND** ("neurogenic shock" OR "spinal cord injury")<br>[neurogenic shock or spinal cord injury]	4
	**891**

In this search strategy, each medical condition was not combined with each other with ‘’OR’’. Instead, keywords associated with each condition were searched with those in [Astronaut and space].

Embase

Num	Search strategy Embase 20180731	Hits
1	astronaut*:ti,ab OR cosmonaut:de,ti,ab OR 'weightlessness':de,ti,ab OR ('space'/de AND 'flight'/de) OR 'space flight'/de OR ((space NEAR/3 (flight OR exploration OR simulation OR missions OR 'long duration' OR deep)):ti,ab) OR spacecraft:ti,ab OR ((analog NEAR/1 environment*):ti,ab) OR 'extraterrestrial life':ti,ab OR moon:de,ti,ab OR astronomy:de,ti,ab OR 'national aeronautics and space administration':ti,ab OR nasa:ti,ab OR 'aerospace medicine':de,ti,ab OR 'environmental medicine':de,ti,ab OR 'space and space related phenomena'/de [Astronaut and space]	57,501
2	atrial fibrillation'/exp OR 'atrial fibrillation*':ti,ab OR 'auricular fibrillation*':ti,ab OR 'atrium fibrillation*':ti,ab OR 'heart attack*':ti,ab OR 'acute coronary syndrome':ti,ab [Atrial fibrillation]	179,768
3	heart infarction'/exp OR ami:ti,ab OR mi:ti,ab OR 'myocardial infarct*':ti,ab OR 'acute heart infarction':ti,ab OR 'coronary thrombos*':ti,ab OR myomala*:ti,ab OR 'acute coronary syndrome*':ti,ab OR 'heart attack*':ti,ab OR 'myocardial isch*':ti,ab OR 'post-infarction':ti,ab [Myocardial infarction]	475,937
4	thromboembolism'/exp OR 'thrombosis'/exp OR 'vein thrombosis'/exp OR 'thrombus'/exp OR 'microthrombus'/exp OR thrombus:ti,ab OR microthrombus:ti,ab OR thrombotic:ti,ab OR thrombilic:ti,ab OR thromboemboli*:ti,ab OR thrombos*:ti,ab OR 'deep vein thrombosis'/exp OR ((('deep vein' OR 'deep venous') NEAR/2 thrombo*):ti,ab) OR dvt:ti,ab OR vte:ti,ab OR 'exp lung' OR 'pulmonary artery'/exp OR 'lung embolism'/exp OR ((pulmonary NEAR/1 embol*):ti,ab) OR ((pulmonary NEAR/1 thrombo*):ti,ab) OR (lung NEAR/1 embol*) OR ((lung NEAR/1 thrombo*):ti,ab) OR pe:ti,ab [Pulmonary embolim]	624,474
5	urolithiasis'/exp OR 'urinary calcul*':ti,ab OR 'bladder calcul*':ti,ab OR 'kidney calcul*':ti,ab OR 'ureteral calcul*':ti,ab OR 'urinary stone*':ti,ab OR 'bladder stone*':ti,ab OR 'kidney stone*':ti,ab OR 'ureteral stone*':ti,ab OR 'urinary colic':ti,ab OR 'bladder colic':ti,ab OR 'renal colic':ti,ab OR 'ureteral colic':ti,ab OR urolithiasis:ti,ab OR ureterolithiasis:ti,ab OR nephrolithiasis:ti,ab OR 'kidney colic'/exp [Nephrolithiasis]	67,482

6	retina tear'/exp OR 'retinal detachment'/exp OR 'vitreous body detachment'/exp OR ((retina$ NEAR/2 break$):ti,ab) OR ((retina$ NEAR/2 tear$):ti,ab) OR ((retina$ NEAR/2 detach$):ti,ab) OR ((retina$ NEAR/2 perforat$):ti,ab) [Retinal detachment]	33,873
7	Sepsis/exp or 'Shock, Septic'/exp or Septicemia/exp OR (sepsis* or septic*):ti,ab OR 'Multiple Organ Failure'/exp OR (multi?organ NEAR/6 failure):ti,ab [Sepsis]	316,543
8	cerebrovascular disease'/exp or 'basal ganglion hemorrhage'/exp or 'brain hematoma'/exp or 'brain hemorrhage'/exp or 'brain infarction'/exp or 'brain ischemia'/exp or 'carotid artery disease'/exp or 'cerebral artery disease'/exp or 'cerebrovascular accident'/exp or 'intracranial aneurysm'/exp or 'occlusive cerebrovascular disease'/exp OR 'stroke patient'/exp or 'stroke unit'/exp OR (stroke or poststroke or post-stroke or cerebrovasc* or 'brain vasc*' or 'cerebral vasc*' or cva* or apoplex* or SAH):ti,ab OR ((brain* or cerebr* or cerebell* or intracran* or intracerebral) NEAR/5 (isch?emi* or infarct* or thrombo* or emboli* or occlus*)):ti,ab OR ((brain* or cerebr* or cerebell* or intracerebral or intracranial or subarachnoid) NEAR/5 (haemorrhage* or hemorrhage* or haematoma* or hematoma* or bleed*)):ti,ab OR 'brain injury'/exp or 'acquired brain injury'/exp OR hemiparesis/exp or hemiplegia/exp or paresis/exp or 'neurologic gait disorder'/exp or 'hemiplegic gait'/exp OR (hempar* or hemipleg* or brain injur*):ti,ab [stroke]	940,278
9	intervertebral disk'/exp OR 'intervertebral disk hernia'/exp OR 'disc herniation':ti,ab OR 'low back pain'/exp [Herniated disk]	82,615
10	cardiogenic shock'/exp OR 'cardiogenic shock':ti,ab [Cardiogenic shock]	25,354
11	('Vision Disorders':ti,ab or 'Vision Disorders'/exp OR 'disabilities, vision':ti,ab OR 'disability, vision':ti,ab OR 'disorder*, visual':ti,ab OR 'impairment*, visual':ti,ab) OR ('Intracranial Hypertension'/exp or 'intracranial pressure'/exp or 'Visual impairment intracranial pressure':ti,ab) [Visual impairment]	250,967
12	intraocular foreign body'/exp OR 'eye injuries, penetrating':ti,ab OR 'eye injury'/exp OR 'eye injuries':ti,ab OR 'eye penetration':ti,ab [Eye penetration]	37,63
13	neurogenic shock'/exp OR 'neurogenic shock':ti,ab	197
14	Spinal Cord Injuries'/exp	70,535
15	Spinal Cord Ischemia'/exp	3,609

16	Central Cord Syndrome'/exp	320
17	(myelopathy NEAR/3 (traumatic or post-traumatic)):ti,ab	127
18	((spine or spinal) NEAR/3 (fracture* or wound* or trauma* or injur* or damag*)):ti,ab	65,717
19	('spinal cord' NEAR/3 (contusion or laceration or transaction or trauma or ischemia)):ti,ab	5,061
20	central cord injury syndrome':ti,ab	1
21	('cervical spine'/exp AND injury/exp)	13,349
22	SPINAL CORD'/exp	100,588
23	SCI:ti,ab	41,515
24	PARAPLEGIA/exp	23,15
25	Quadriplegia/exp	15,928
26	(paraplegia* or quadriplegia* or tetraplegia*):ti,ab	21,145
27	distributive shock':ti,ab OR 'neurogenic vasoplegia':ti,ab OR neurogenic:ti,ab OR hypotension:ti,ab	105,579
28	(hypotension OR 'distributive shock' OR 'neurogenic vasoplegia' OR 'neurogenic hypotension'):ti,ab	70,37
29	13 -27/OR [Neurogenic shock- spinal cord injury]	344,055
30	2 - 12/OR	2,524,793
31	12 OR 28	2,805,306
32	30 AND 1	**3,148**

IeeeXplore

**IEEE Xplore- 20180731**	Syntax to be used with keywords	Hits to retrieve
astronauts	(("Document Title": Astronauts OR "Document Title": space shuttles OR "Document Title": space exploration OR "Document Title": Planets OR "Document Title": Space flight OR "Document Title": deep space OR "Document Title": long-duration space exploration missions OR "Document Title": NASA))<br>4149 Hits	
Atrial fibrillation	"Document Title": Atrial fibrillation OR "Publication Title": Atrial fibrillation OR "Abstract": Atrial fibrillation OR Index Terms: Atrial fibrillation OR "Document Title": Atrial flutter OR "Publication Title": atrial flutter OR "Abstract": atrial flutter OR Index Terms: atrial flutter OR "Document Title": Cardiac Arrhythmias OR "Publication Title": Cardiac Arrhythmias OR "Abstract": Cardiac Arrhythmias OR Index Terms: Cardiac Arrhythmias<br><br>2489 Hits	2504
myocardial infarction	"Document Title":myocardial infarction OR "Publication Title": myocardial infarction OR "Abstract": myocardial infarction OR Index Terms: myocardial infarction OR "Document Title": heart infarction OR "Publication Title": heart infarction OR "Abstract": heart infarction OR Index Terms: heart infarction<br><br>970 Hits	24

Pulmonary embolism	"Document Title": Pulmonary embolism OR "Publication Title": Pulmonary embolism OR "Abstract": Pulmonary embolism OR Index Terms: Pulmonary embolism OR "Document Title": Pulmonary Thromboembolism OR "Publication Title": Pulmonary Thromboembolism OR "Abstract": Pulmonary Thromboembolism OR Index Terms: Pulmonary Thromboembolism<br><br>60 Hits	4
Nephrolithiasis	"Document Title": Nephrolithiasis OR "Publication Title": Nephrolithiasis OR "Abstract": Nephrolithiasis OR Index Terms: Nephrolithiasis OR "Document Title": renal colic OR "Publication Title": renal colic OR "Abstract": renal colic OR Index Terms: renal colic OR "Document Title": Urolithiasis OR "Publication Title": Urolithiasis OR "Abstract": Urolithiasis OR Index Terms:Urolithiasis<br><br>10 Hits	0
retinal detachment	"Document Title": retinal detachment OR "Publication Title": retinal detachment OR "Abstract": retinal detachment OR "Document Title": retinal perforation OR "Publication Title": retinal perforation OR "Abstract": retinal perforation<br><br>24 Hits	1

sepsis	"Document Title": sepsis OR "Publication Title": sepsis OR "Abstract": sepsis OR "Document Title": septicemia OR "Publication Title": septicemia OR "Abstract": septicemia<br><br>124 Hits	2
stroke	"Document Title": stroke OR "Publication Title": stroke OR "Abstract": stroke OR "Document Title": cerebrovascular disorders OR "Publication Title": cerebrovascular disorders OR "Abstract": cerebrovascular disorders<br><br>9824 hits	552
Intervertebral Disc	"Document Title": Intervertebral Disc OR "Publication Title": Intervertebral Disc OR "Abstract": Intervertebral Disc OR "Document Title": herniated disk OR "Publication Title": herniated disk OR "Abstract": herniated disk<br><br>145 Hits	8
Cardiogenic shock	"Document Title": Cardiogenic shock OR "Publication Title": Cardiogenic shock OR "Abstract": Cardiogenic shock<br><br>9 hits	40

Vision Disorders	("Document Title": Vision Disorders OR "Publication Title": Vision Disorders OR "Abstract": Vision Disorders OR "Document Title": VIIP OR "Publication Title": VIIP OR "Abstract": VIIP) AND ("Document Title": intracranial pressure OR "Publication Title":intracranial pressure OR "Abstract": intracranial pressure)<br><br>2 hits	11
Penetrating Eye Injuries	("Document Title": Penetrating Eye Injuries OR "Publication Title": Penetrating Eye Injuries OR "Abstract": Penetrating Eye Injuries OR "Document Title": Eye Foreign Bodies OR "Publication Title": Eye Foreign Bodies OR "Abstract": Eye Foreign Bodies)<br><br>192 hits	15
neurogenic shock spinal cord injury	"Document Title": neurogenic shock OR "Publication Title": neurogenic shock OR "Abstract": neurogenic shock OR "Document Title": spinal cord injury OR "Publication Title": spinal cord injury OR "Abstract": spinal cord injury<br><br>985 hits	51

Using a complex strategy was not possible in this database, which is why keywords from each medical condition were combined with the following space-related keywords separately: aerospace, Aerospace Medicine, analog environment, astronaut, Cosmonaut, countermeasure, deep space, ecological system, environmental medicine, Extraterrestrial Environment, long-duration space exploration missions, NASA, Planets, spacecraft, space exploration, space flight, space shuttles, Space Simulation, United States National Aeronautics and Space Administration, Weightlessness. The hits to review column represent the total number of articles found after summing the number of hits from each combination per medical condition.

Medline Ovid

Num	**Medline OVID 20180731**	Hits
1	"astronaut*".ab,ti. or astronauts/ or "Cosmonaut*".ab,ti. or Weightlessness/ or Weightlessness.ab,ti. or Space Flight/ or spacecraft.ab,ti. or spacecraft/ or Long-duration space exploration missions.ab,ti. or Space Simulation/ or aerospace.ab,ti. or "analog environment*".ab,ti. or deep space.ab,ti. or "ecological system*".ab,ti. or Ecological Systems, Closed/ or extraplanetary.ab,ti. or extraterrestrial.ab,ti. or Extraterrestrial Environment/ or planets/ or countermeasure.ab,ti. or countermeasure.ab,ti. or Weightlessness Countermeasures/ or "United States National Aeronautics and Space Administration"/ or Aerospace Medicine.ab,ti. or Aerospace Medicine/ or environmental medicine/ or environmental medicine.ab,ti. or Space medicine.ab,ti. [Astronaut and space]	41351
2	(Atrial Fibrillation/) OR (atrial fibrillation*.tw.) OR (auricular fibrillation*.tw.) OR (atrium fibrillation*.tw.) OR (Catheter Ablation/) OR (atrial ablation*.tw.) OR ((electric* adj2 ablation*).tw.) OR (catheter ablation*.tw.) OR ((radiofrequency adj2 ablation*).tw.) OR (pulmonary vein isolation*.tw.) [Atrial fibrillation]	94612
3	(exp Myocardial Infarction/) OR (exp Myocardial Ischemia/) OR (ami.tw.) OR (mi.tw.) OR (myocardial infarct$.tw.) OR (acute heart infarction.tw.) OR (coronary thrombos$.tw.) OR (myomala$.tw.) OR (acute coronary syndrome$.tw.) OR (heart attack$.tw.) OR (myocardial isch$.tw.) OR (post-infarction.tw.) [Myocardial infarction]	481243
4	(Thromboembolism/) OR (Venous Thromboembolism/) OR (Thrombosis/) OR (Venous Thrombosis/) OR (Upper Extremity Deep Vein Thrombosis/) OR ((thromboemboli$ or microthrombus or thrombus or thrombo$ or thrombilic or thrombotic).ab,ti) OR ((DVT or VTE).ab,ti) OR (Pulmonary Embolism/) OR ((pulmonary adj embol$).ab,ti) OR ((pulmonary adj thrombo$).ab,ti) OR ((lung adj embol$).ab,ti) OR ((lung adj thrombo$).ab,ti) OR (PE.ab,ti) [Pulmonary embolim]	430796

5	exp urinary calculi/ OR (urinary calcul$ or bladder calcul$ or kidney calcul$ or ureteral calcul$ or ureteric calcul$).tw. OR (urinary stone$ or bladder stone$ or kidney stone$ or ureteral stone$).tw. OR (urinary colic or bladder colic or kidney colic or ureteral colic).tw. OR exp Nephrolithiasis/ OR urolithiasis.tw. OR ureterolithiasis.tw. OR nephrolithiasis.tw. [Nephrolithiasis]	42603
6	(exp retinal detachment/) OR (exp retinal perforation/) OR (exp vitreous detachment/) OR ((retina$ adj2 break$).tw.) OR ((retina$ adj2 tear$).tw.) OR ((retina$ adj2 detach$).tw.) OR ((retina$ adj2 perforat$).tw.) [Retinal detachment]	28583
7	(exp Sepsis/ or exp Shock, Septic/) OR (Systemic Inflammatory Response Syndrome/) OR (Multiple Organ Failure/) OR ((multi?organ adj5 failure).mp.) OR (SIRS.mp.) [Sepsis]	127751
8	(cerebrovascular disorders/ or exp basal ganglia cerebrovascular disease/ or exp brain ischemia/ or exp carotid artery diseases/ or exp intracranial arterial diseases/ or exp intracranial arteriovenous malformations/ or exp "intracranial embolism and thrombosis"/ or exp intracranial hemorrhages/ or stroke/ or exp brain infarction/ or stroke, lacunar/ or vasospasm, intracranial/ or vertebral artery dissection/ or brain injuries/ or brain injury, chronic/) OR (stroke or poststroke or post-stroke or cerebrovasc$ or brain vasc$ or cerebral vasc$ or cva$ or apoplex$ or SAH).tw. OR ((brain$ or cerebr$ or cerebell$ or intracran$ or intracerebral) adj5 (isch?emi$ or infarct$ or thrombo$ or emboli$ or occlus$)).tw. OR ((brain$ or cerebr$ or cerebell$ or intracerebral or intracranial or subarachnoid) adj5 (haemorrhage$ or hemorrhage$ or haematoma$ or hematoma$ or bleed$)).tw. OR (hemiplegia/ or exp paresis/) OR (hempar$ or hemipleg$ or brain injur$).tw. [stroke]	550400
9	Intervertebral Disc/ or Intervertebral Disc Displacement/ or disc herniation.mp. or Low Back Pain/ [Herniated disk]	46082
10	Shock, Cardiogenic/ or Cardiogenic shock.ab,ti. [Cardiogenic shoc]	12737

11	(Vision Disorders.mp. or Vision Disorders/ OR disabilities, vision.mp. OR disability, vision.mp. OR disorder*, visual.mp. OR impairment*, visual.mp.) OR (Intracranial Hypertension/ or intracranial pressure/ or Visual impairment intracranial pressure.mp.) [Vision Disorder]	43281
12	Eye Foreign Bodies/ or Eye Injuries, Penetrating/ or Eye Injuries/ or eye penetration.mp. [Eye penetration]	17023
13	neurogenic shock.mp.	104
14	Spinal Cord Injuries/	34360
15	Spinal Cord Ischemia/	1267
16	Central Cord Syndrome/	89
17	(myelopathy adj3 (traumatic or post-traumatic)).ab,ti.	106
18	((spine or spinal) adj3 (fracture* or wound* or trauma* or injur* or damag*)).ab,ti.	51237
19	(spinal cord adj3 (contusion or laceration or transaction or trauma or ischemia)).ab,ti.	4083
20	central cord injury syndrome.ab,ti.	1
21	central spinal cord syndrome.ab,ti.	9
22	Cervical Vertebrae/in	6166
23	Spinal Cord/	76222
24	SCI.ab,ti.	30229
25	Paraplegia/	12340
26	Quadriplegia/	7711
27	(paraplegia* or quadriplegia* or tetraplegia*).ab,ti.	16169
28	hypotension.ab,ti OR distributive shock.ab,ti OR neurogenic vasoplegia.ab,ti OR neurogenic hypotension.ab,ti	49549
29	OR /13-28 [Neurogenic shock] [Spinal cord injury]	215181
30	#2 OR #3 OR #4 OR #5 OR #6 OR #7 OR #8 OR #9 OR #10 OR #11 OR #12 OR #29	1860337
31	#1 AND #30	**1828**

PsychINFO

Num	**PsychInfo Ovid 20180731**	Hits
1	((astronauts/ or aerospace personnel/ or aircraft pilots/ or spacecraft/ or weightlessness/ or gravitational effects/ or spaceflight/ or Long-duration space exploration missions.ab,ti. or "analog environment*".ab,ti. or deep space.ab,ti. or extraterrestrial.ab,ti. or Extraterrestrial Environment.ab,ti. or planet.ab,ti. or countermeasure.ab,ti. or Weightlessness Countermeasures.ab,ti. or United States National Aeronautics.mp.) and Space Administration.ab,ti.) or NASA.ab,ti. or Aerospace Medicine.ab,ti. or environmental medicine.ab,ti. or Space medicine.ab,ti. [Astronaut and space]	715
2	Myocardial Infarctions/ or coronary thromboses/ or ami.tw. or mi.tw. or myocardial infarct$.tw. or heart infarction.tw. or coronary thrombos$.tw. or myomala$.tw. or coronary syndrome$.tw. or heart attack$.tw. or post-infarction.tw.<br>[Myocardial infarction]	10229
3	Atrial Fibrillation/ or atrial fibrillation*.tw. or auricular fibrillation*.tw. or atrium fibrillation*.tw. or Catheter Ablation/ or atrial ablation*.tw. or (electric* adj2 ablation*).tw. or catheter ablation*.tw. or (radiofrequency adj2 ablation*).tw. or pulmonary vein isolation*.tw.<br>[Atrial fibrillation]	1385
4	(Thromboembolism/) OR (Venous Thromboembolism/) OR (Thrombosis/) OR (Venous Thrombosis/) OR (Upper Extremity Deep Vein Thrombosis/) OR ((thromboemboli$ or microthrombus or thrombus or thrombo$ or thrombilic or thrombotic).ab,ti) OR ((DVT or VTE).ab,ti) OR (Pulmonary Embolism/) OR ((pulmonary adj embol$).ab,ti) OR ((pulmonary adj thrombo$).ab,ti) OR ((lung adj embol$).ab,ti) OR ((lung adj thrombo$).ab,ti) OR (PE.ab,ti)<br>[Pulmonary embolim]	7858
5	(urinary calcul$ or bladder calcul$ or kidney calcul$ or ureteral calcul$ or ureteric calcul$).tw. OR (urinary stone$ or bladder stone$ or kidney stone$ or ureteral stone$).tw. OR (urinary colic or bladder colic or kidney colic or ureteral colic).tw. OR exp Nephrolithiasis/ OR urolithiasis.tw. OR ureterolithiasis.tw. OR nephrolithiasis.tw.<br>[Nephrolithiasis]	105
6	(exp retinal perforation/) OR (exp vitreous detachment/) OR ((retina$ adj2 break$).tw.) OR ((retina$ adj2 tear$).tw.) OR ((retina$ adj2 detach$).tw.) OR ((retina$ adj2 perforat$).tw.)<br>[Retinal detachment]	111

7	sepsis/ or sepsis.ab,ti. or blood poisoning*.mp. or pyaemia*.mp. or pyemias.mp. or sepsis, severe.mp. or septicemias.mp.<br>[Sepsis]	804
8	stroke.mp. or stroke/ or stroke*.mp. or acute cerebrovascular accident*.mp. or cva*.mp. or vascular accident, brain.ab,ti. or vascular accident, brain.mp. or apoplexy.mp. or apoplexy, cerebrovascular.mp. or brain vascular accident*.mp.<br>[stroke]	34195
9	Intervertebral Disc/ or Intervertebral Disc Displacement/ or disc herniation.mp. or Low Back Pain/<br>[Herniated disk]	114
10	Shock, Cardiogenic/ or Cardiogenic shock.ab,ti.<br>[Cardiogenic shoc]	26
11	Vision Disorders.mp. or Vision Disorders/ or disabilities, vision.mp. or disability, vision.mp. or disorder*, visual.mp. or impairment*, visual.mp.<br>[Vision Disorder]	6950
12	(Eye Foreign Bodies or Eye Injuries or eye penetration).mp.<br>[Eye penetration]	42
13	(neurogenic shock or sipnal cord injury).mp.<br>[Neurogenic shock] [Spinal cord injury]	5
14	#2 or #3 or #4 or #5 or #6 or #7 or #8 or #9 or #10 or #11 or #12 or #13	57405
15	#1 and #14	**7**

Web of Science

Num	Web of science 20180731	Hits
1	astronaut* or Cosmonaut*or Weightlessness or Space Flight or spacecraft or Long-duration space exploration missions or Space Simulation or aerospace or "analog environment*"or deep space or "ecological system*" or Ecological Systems, Closed/ or extraplanetary or extraterrestrial or Extraterrestrial Environment or planets or countermeasure or Weightlessness Countermeasures or "United States National Aeronautics and Space Administration"/ or Aerospace Medicine or environmental medicine/ or environmental medicine or Space medicine [Astronaut and space]	386,707
2	Atrial fibrillation OR atrial flutter or Cardiac Arrhythmias [Atrial fibrillation]	120,854
3	myocardial infarct* or heart infarct* or coronary thrombosis or coronary syndrome or heart attack or post-infarction or AMI [Myocardial infarction]	354,966
4	Pulmonary embolism*OR pulmonary thromboembolism OR embolism* [Pulmonary embolim]	56,197
5	Nephrolithiasis OR Urolithiasis OR Renal Colic [Nephrolithiasis]	14,285
6	retinal detachment OR retinal perforation OR vitreous detachment [Retinal detachment]	16,186
7	(sepsis* or septic* or SIRS) or (Systemic Inflammatory Response Syndrome) or (Multiple Organ Failure) or (multi?organ SAME failure) [Sepsis]	179,818
8	Stroke OR stroke* OR acute cerebrovascular accident* OR cva*or vascular accident, brain or vascular accident, brain or apoplexy or apoplexy, cerebrovascular or brain vascular accident* [stroke]	293,318
9	Intervertebral Disc OR Intervertebral Disc Displacement OR disc herniation OR Low Back Pain [Herniated disk]	57,378
10	Cardiogenic shock [Cardiogenic shoc]	9,864
11	(Vision Disorders OR disabilities, vision OR disability, vision OR disorder*, visual OR impairment*, visual) OR (Intracranial Hypertension OR intracranial pressure OR Visual impairment intracranial pressure) [Vision Disorder]	84,57
12	Eye Foreign Bodies OR Penetrating Eye Injuries OR Eye Injuries OR eye penetration [Eye penetration]	13,208
13	neurogenic shock OR Spinal Cord Injuries OR Spinal Cord Ischemia OR Central Cord Syndrome OR spinal fracture OR spinal wound, or spinal trauma OR central cord injury syndrome OR (paraplegia* or quadriplegia* or tetraplegia*) OR hypotension OR distributive shock OR neurogenic vasoplegia OR neurogenic hypotension [Neurogenic shock]	132,725

14	#2 OR #3 OR #4 OR #5 OR #6 OR #7 OR #8 OR #9 OR #10 OR #11 OR #12 OR #13	1,207,643
15	#1 AND #14	**3,113**
